# Real-time determination of enantiomeric and isomeric content using photoelectron elliptical dichroism

**DOI:** 10.1038/s41467-018-07609-9

**Published:** 2018-12-06

**Authors:** A. Comby, E. Bloch, C. M. M. Bond, D. Descamps, J. Miles, S. Petit, S. Rozen, J. B. Greenwood, V. Blanchet, Y. Mairesse

**Affiliations:** 1 Université de Bordeaux - CNRS - CEA, CELIA, UMR5107, F33405 Talence, France; 2 School of Maths and Physics, Queen’s University, Belfast, BT7 INN UK; 3 Weizmann Institute of Science, Rehovot, 76100 Israel

## Abstract

The fast and accurate analysis of chiral chemical mixtures is crucial for many applications but remains challenging. Here we use elliptically-polarized femtosecond laser pulses at high repetition rates to photoionize chiral molecules. The 3D photoelectron angular distribution produced provides molecular fingerprints, showing a strong forward-backward asymmetry which depends sensitively on the molecular structure and degree of ellipticity. Continuously scanning the laser ellipticity and analyzing the evolution of the rich, multi-dimensional molecular signatures allows us to observe real-time changes in the chemical and chiral content present with unprecedented speed and accuracy. We measure the enantiomeric excess of a compound with an accuracy of 0.4% in 10 min acquisition time, and follow the evolution of a mixture with an accuracy of 5% with a temporal resolution of 3 s. This method is even able to distinguish isomers, which cannot be easily distinguished by mass-spectrometry.

## Abstract (web summary)

The analysis of chiral chemical mixtures is crucial for many applications. Here, the authors perform real-time analysis of samples by ionizing them with elliptically polarized femtosecond laser pulses and detecting the angular distributions of the photoelectrons.

## Introduction

Chiral molecules are not superimposable onto their mirror image, and can only be distinguished through their interaction with another chiral object. Since all living organisms are constituted of chiral building blocks, chirality plays a crucial role in biology and biochemistry, and therefore in industrial sectors such as pharmaceuticals, food, or agro-chemistry for instance. The relative proportion of the two mirror images of chiral molecules (for instance, enantiomers *R* and *S*) in a sample is called the enantiomeric excess and is defined as *ee* = ([*R*] − [*S*])/([*R*] + [*S*]). Its fast and accurate determination is of prime importance for many applications, particularly in the context of increasingly stringent legislation.

One of the most common techniques used to identify chiral molecules is chromatography. The sample to be analyzed is sent into a column containing chiral selectors which interact differently with the two enantiomers. As a consequence, the two enantiomers have different travel times through the column and can be separated. In the gas phase, the state-of-the-art in *ee* determination is GCxGC-TOFMS (two-dimensional gas chromatography–time-of-flight mass spectrometry), which reaches 0.1% range accuracies by combining two successive chiral columns with mass spectrometry detection^1^. Its main drawbacks are the lack of a universal chiral column which is able to separate all classes of chiral compounds, the limited lifetime of the columns, and the necessity to fine tweak the temperature and pressure for each analyzed sample. In addition, in the gas phase the measurement speed is intrinsically limited by the migration time, such that measurements typically take several tens of minutes, and are incompatible with continuous monitoring of enantiomeric excess.

To speed up measurements, it is necessary to use a physical process which is faster than the migration on a substrate. This is naturally achieved in chiroptical measurements, which rely on the interaction of circularly polarized electromagnetic radiation with chiral molecules and whose response time is on the attosecond range^2,3^. Absorption circular dichroism (CD), which measures the different absorption of left and right circularly polarized light in a sample, played an important role historically. Its interest was renewed in the 1970s when infrared CD revealed that transitions between vibrational states provided a unique chiral fingerprint, with unambigious determination of the absolute configuration when compared to ab initio calculations^4–6^. This effect, called vibrational circular dichroism (VCD), is now used in commercial instruments to analyze chiral samples with high accuracy (~1%), and enables enantiomeric excesses to be monitored in real time^7,8^. On the other hand, VCD remains a rather weak effect, relying on electric quadrupole and magnetic dipole transitions. This means that VCD is only performed in the condensed phase, requiring a large amount of molecules.

As reviewed in ref. ^9^, chiroptical techniques have emerged in the past few years, which offer unprecedented sensitivities: photoelectron circular dichroism^10–15^, microwave spectroscopy^16–18^, photoexcitation circular dichroism^19^, and chiral high-order harmonic generation^2^. In microwave spectroscopy, the free-induction decay of a rotational transition excited by a combination of two fields with different polarization directions provides an enantiosensitive signal^16^, which can be used to determine *ee* with demonstrated accuracies of 1% in 90 s^17^ and to distinguish conformers, isomers, or isotopologues^9,18^. Photoelectron circular dichroism (PECD) was also shown to be very promising for chiral analysis. PECD occurs when gas-phase chiral molecules are photoionized by circularly polarized light. More electrons are emitted forward or backward relative to the light propagation axis, depending on the handedness of the light and of the enantiomer^10,11^. PECD is a pure electric-dipole effect, leading to very large signals (in the 1–10% range)^14^. PECD was thus used to determine enantiomeric excess with accuracies better than 1% in a few hours using extreme ultraviolet (XUV) synchrotron radiation^20^. Using ultraviolet (UV)–visible femtosecond lasers, accuracies better than 1% were achieved in around 10 min^21^, and 5% in 1 min using a high repetition rate laser^22^. PECD was also associated with coincidence electron-ion detection to analyze multi-component mixtures, distinguishing molecules with an accuracy of 20% in 18 h acquisition time^23^.

Here we introduce a way of determining enantiomeric excesses through photoelectron elliptical dichroism. We show that the resonance-enhanced multiphoton ionization (REMPI) of chiral molecules by elliptically polarized laser pulses produces strong forward–backward asymmetries in the three-dimensional (3D) photoelectron angular distributions which can evolve non-monotonically with the ellipticity of the laser light. We continuously record the forward–backward asymmetry while modulating periodically the laser ellipticity, and show that the resulting signal enables the fast and accurate determination of enantiomeric excesses, with a 0.4% accuracy in 10 min and 5% accuracy in 3 s. This breakthrough enables real-time tracking of the composition of chiral samples. Lastly, we demonstrate that photoelectron elliptical dichroism can be used to identify different chemical species in a mixture without the need of mass spectrometry, and can even distinguish isomers.

## Results

### Photoelectron elliptical dichroism

To perform accurate photoionization measurements, we used the Blast Beat laser system at CELIA, whose repetition rate can be tuned between 166 kHz and 2 MHz (see Methods). A 515 nm beam, obtained by frequency doubling the 1030 nm 130 fs pulses, was focused into the interaction chamber of a Cold Target Recoil Ion Momentum Spectrometer (COLTRIMS), where it photoionized enantiopure fenchone molecules. The laser polarization state was controlled by rotating a half waveplate in front of a fixed quarter waveplate. This enables scanning the ellipticity while keeping a fixed direction of the ellipse. Throughout this study, the ellipticity of the ionizing laser pulse will be quantified by the third Stokes parameter *S*_3_, which represents the amount of circularly polarized light in the radiation. The COLTRIMS provides a complete map of the 3D photoelectron angular distribution in momentum space *P*(p_*x*_, *p*_*y*_, *p*_*z*_)^24^. The distributions obtained by photoionizing fenchone with right and left polarized light *P*_*R*_(*p*_*x*_, *p*_*y*_, *p*_*z*_) and *P*_*L*_(*p*_*x*_, *p*_*y*_, *p*_*z*_) were used to produce two 3D distributions: the photoelectron angular distribution 3*D* − *PAD* = (*P*_*L*_ + *P*_*R*_)/2, and the photoelectron elliptical dichroism (PEELD) 3*D* − *PEELD* = (*P*_*L*_ − *P*_*R*_). These two distributions are expected to be respectively symmetric and antisymmetric with respect to the laser propagation axis *z*. In practice, we symmetrize and antisymmetrize them to correct for artifacts due to the imperfect nature of the waveplate and the inhomogeneities of the detector.

**Fig. 1:**
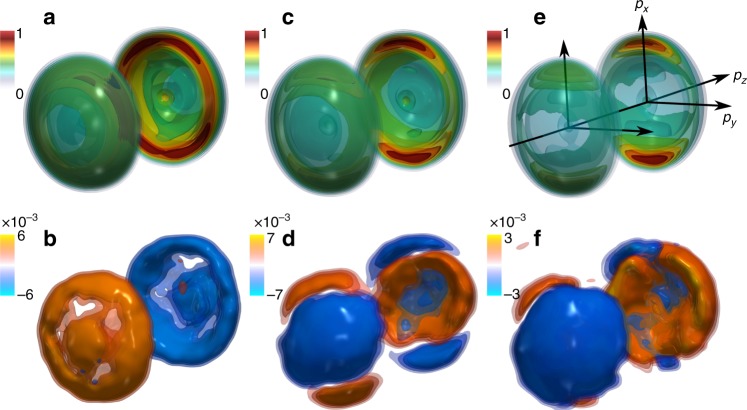
Photoelectron angular distributions and elliptical dichroism. The 3D photoelectron angular distributions (top) and photoelectron elliptical dichroism (bottom) produced by photoionizing (+)-fenchone molecules at 6 × 10^12^ W cm^−2^, and detected using a COLTRIMS instrument. The Stokes parameter *S*_3_ of the ionizing laser pulses are 1 (**a**, **b**), 0.9 (**c**, **d**), and 0.6 (**e**, **f**). The 3D-PEELDs are normalized to the maximum of the 3D-PAD

Figure 1 shows isosurface plots of the photoelectron distributions, for different laser ellipticities. When the laser light is circularly polarized (*S*_3_ = 1), the distributions show an almost perfect cylindrical symmetry around *p*_*z*_ (Fig. 1a). The 3D-PAD and 3D-PEELD peak around 0.25 atomic units (a.u.) momentum (0.88 eV energy), which is characteristic of photoelectrons emitted from the highest occupied molecular orbital (HOMO, ionization potential 8.72 eV) by 4 photon ionization at 515 nm. This ionization process is enhanced by a resonance at 7.2 eV (3 + 1 REMPI). The 3D-PEELD is dominantly negative in the forward direction and positive backwards. When *S*_3_ decreases by 10%, the overall 3D-PEELD changes sign, and lobes of opposite sign appear (Fig. 1d). The 3D-PAD sharpens around the direction of the main axis of the laser polarization ellipse (Fig. 1c). A twist appears in the 3D-PEELD in the *p*_*x*_,*p*_*y*_ plane, and is enhanced when *S*_3_ further decreases to reach *S*_3_ = 0.6 (Fig. 1f). These measurements show that the forward–backward photoelectron asymmetry produced by REMPI of chiral molecules can switch sign as soon as the light becomes slightly elliptical. The fast and accurate determination of enantiomeric excesses that will be presented later is built upon this elliptical dependency of the REMPI-PEELD.

**Fig. 2:**
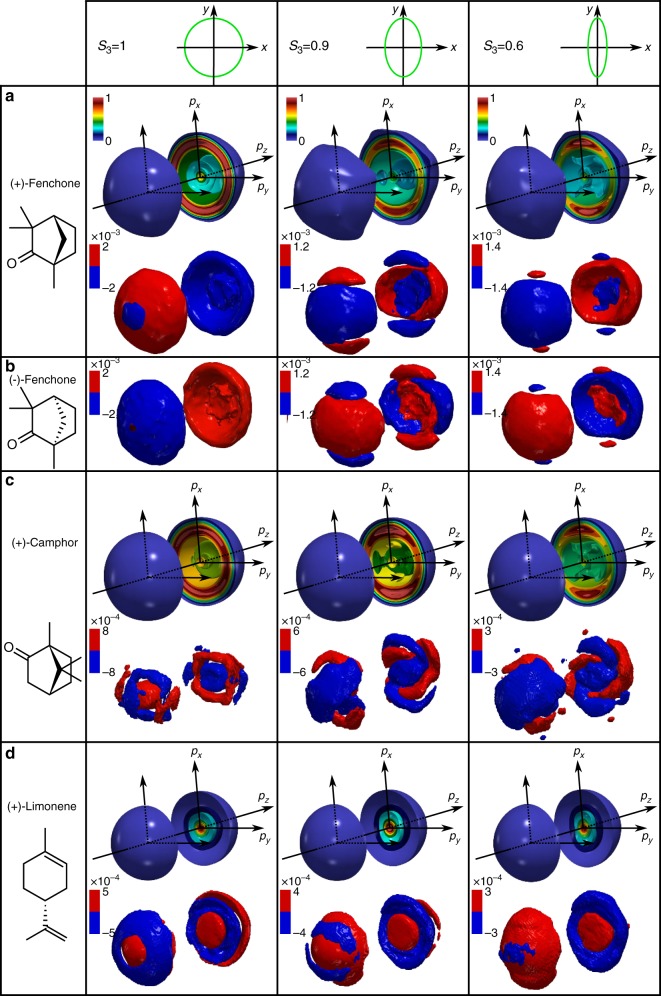
The 3D-PAD and 3D-PEELD recorded by VMI tomography. Results obtained by photoionizing (+)-fenchone (**a**), (−)-fenchone (**b**), (+)-camphor (**c**), and (+)-limonene (**d**) with 515 nm pulses at 5 × 10^12^ W cm^−2^. The images are reconstructed from 36 tomographic projections recorded by rotating the laser ellipse by steps of 10° around the *z*-axis. The Stokes parameter *S*_3_ of the ionizing laser pulses are 1 (left), 0.9 (middle), and 0.6 (right). The 3D-PEELDs are normalized to the maximum of the 3D-PAD

The COLTRIMS detection has the advantage that it directly measures the 3D-PADs, but is restricted to around one event per laser shot to maintain a precise determination of the arrival time of the photoelectron on the detector. Furthermore, it is a costly and complex device, which is unlikely to be deployed widely for practical applications in analytical chemistry. The setup can be simplified by combining a velocity map imaging (VMI) spectrometer which records only the (*x*, *z*) projection of the 3D-PAD^25^, with a tomographic procedure in which the 3D-PAD is rotated along the *z*-axis by rotating the laser ellipse^26,27^. Higher acquisition speeds can be achieved with such a setup. For (+)-fenchone the results presented in Fig. 2 show very good agreement with the COLTRIMS measurements, with a 4 times shorter acquisition time, validating the procedure.

In order to evaluate the generality of the effect, we repeated the measurements in other molecules. Figure 2 demonstrates that in multiphoton ionization, the enhanced sensitivity of PEELD compared to PECD is not restricted to fenchone, but also exists in camphor and limonene. The results also show that PEELD is extremely sensitive to isomerism: camphor and fenchone only differ by the position of two methyl groups that are not attached to asymmetric carbons, have similar ionization potentials, and show very similar 3D-PADs. However, they generate very different 3D-PEELDs. This can be seen as an extension of the isomerism sensitivity of PECD, observed both in single-photon^20,28^ and multiphoton ionization^12^. The difference between PECD and PEELD is spectacular in camphor, where a clear twist is observed in an elliptical ionizing field. Interestingly, the direction of this twist is imposed by the enantiomer, as can be seen in the comparison of measurements performed in (+) and (−)-fenchone. In limonene the 3D-PEELD distributions remain much more symmetric but show energy components with opposite signs, as is often observed in multiphoton ionization^29^. The outer component disappears when the laser ellipticity decreases.

**Fig. 3:**
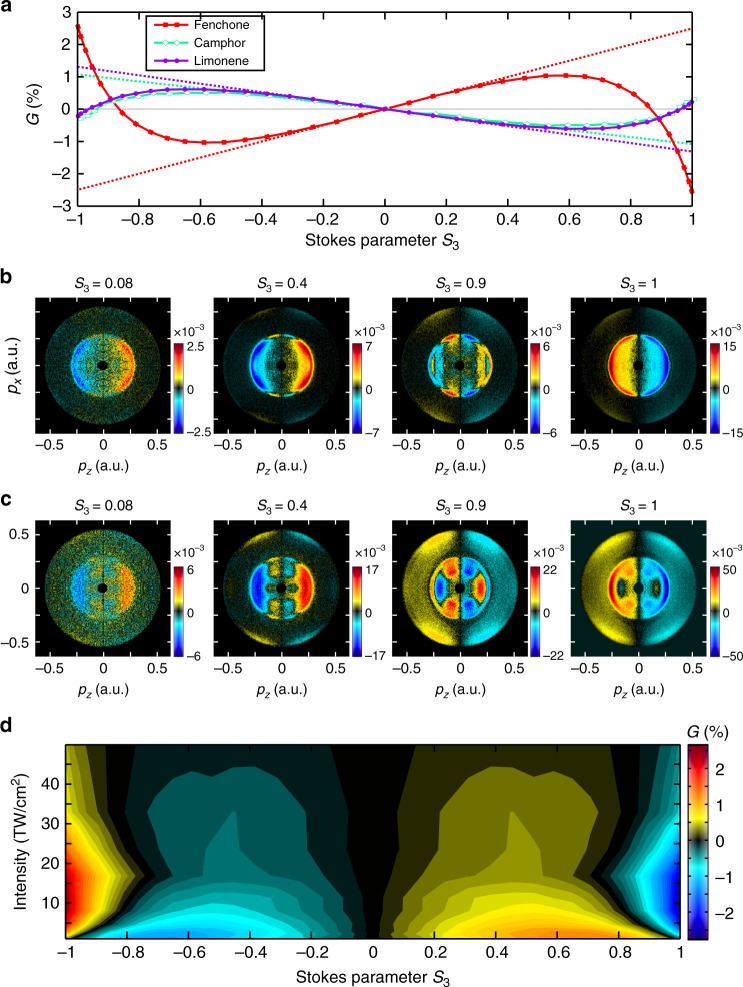
Ellipticity and intensity dependence of PEELD. **a** Evolution of the the forward/backward asymmetry *G* as a function of *S*_3_, in (+)-fenchone (red squares), (+)-camphor (green diamonds), and (+)-limonene (purple circles), at 5 × 10^12^ W cm^−2^. The dotted lines are a linear extrapolation of the low-*S*_3_ behavior. **b**, **c** Projections of the 3D-PEELD from (+)-fenchone in the (*p*_*x*_, *p*_*z*_) plane, at 5 × 10^12^ W cm^−2^ (**b**) and 1.7 × 10^13^ W cm^−2^ (**c**). The *z*-axis is the laser propagation direction and the *x*-axis is the main axis of the laser ellipse, and the VMI detector lies in the (*x*, *z*) plane. The (*p*_*x*_, *p*_*z*_) projection are integration over the time-of-flight (*y*) axis of the VMI. **d** Evolution of the ellipticity dependence of the forward/backward asymmetry *G* with laser intensity in (+)-fenchone

The 3D isosurface maps of the PEELD enable the angular and kinetic energy dependence of the process to be resolved but make the estimation of the overall forward–backward asymmetry quite difficult. We thus calculated the asymmetry factor usually defined in PECD studies as twice the difference between the number of electrons emitted in the forward *F* and backward *B* hemispheres, normalized by the mean signal per hemisphere: *G* = 4(*F* − *B*)/(*F* + *B*). The evolution of *G* as a function of *S*_3_ is shown in Fig. 3a, at an intensity of 1 × 10^13^ W cm^−2^. When the light is mostly linearly polarized, the asymmetry increases linearly with *S*_3_. When *S*_3_ reaches ~0.6, the asymmetry decreases, changes sign, and maximizes in circular polarization (*S*_3_ = ±1). In fenchone the overall maximum of the forward/backward electron emission is reached when the ionizing radiation is circular, but in camphor and limonene the asymmetry is higher in elliptical light, around *S*_3_ ~ ±0.6.

The ellipticity dependence of PECD has been investigated in previous work, but the key features discussed here had not been observed. In the single-photon vacuum UV (VUV) ionization regime, the forward/backward asymmetry in the photoelectron ejection scales linearly with the amount of circularly polarized light *S*_3_^30^. In the multiphoton ionization of camphor by 400 nm pulses, Lux et al.^27^ observed a monotonic increase of the asymmetry, with an increasing slope around very high ellipticities. At the same wavelength with lower laser intensity (4 × 10^10^ W cm^−2^) Miles et al.^22^ reported a similar observation, but found a clear slope change around |*S*_3_| ~0.4.

What is the origin of the non-monotonic behavior of *G* with laser ellipticity? In the three molecular species considered here, the resonant absorption of three photons at 515 nm (2.4 eV) promotes the molecules to high-lying Rydberg states, from which they are photoionized by further absorption of one or several photons. By decoupling the excitation and ionization steps through a two-color experiment, we recently demonstrated that photoexciting molecules with linearly or circularly polarized photons led to strong changes in the PECD, with possible sign inversions^31^. This is the result of the anisotropy of excitation introduced by the resonance. The multiphoton excitation from the ground to the Rydberg states breaks the isotropy of the sample by preferentially exciting molecules whose transition dipole moment is parallel to the laser field. Thus, circularly polarized radiation tends to promote molecules whose transition dipole moment lies in the polarization plane, while linearly polarized light selects the molecules whose transition dipole moment is parallel to the polarization axis. Since PECD strongly depends on the molecular orientation^32,33^, the different molecular orientations selected through photoexcitation can have a strong influence on the resulting PECD.

Here we lie in an intermediate regime in which the ionizing field is elliptically polarized. This field can be decomposed as a sum of linear and circular components, the latter being proportional to *S*_3_. When *S*_3_ is small the intensity of the linear components is much stronger than that of the circular part. In addition, linearly polarized light is more efficient for multiphoton excitation than circular radiation. Thus, the multiphoton transition to the Rydberg states most likely involves three linearly polarized photons. From this Rydberg state the molecules can be ionized by absorption of a linear photon, leading to no dichroism, or of a circular photon, producing a forward–backward photoelectron asymmetry. This 3 + 1 resonant enhanced multiphoton ionization (REMPI) can thus be seen as a single-photon PECD experiment, in which the ionized system is an ensemble of orientated, photoexcited molecules. In this regime the dichroism scales linearly with the amount of circularly polarized light *S*_3_, as established in the VUV range^30^. Our experimental results indicate that this picture is correct in the three investigated molecules as long as |*S*_3_| <0.4. This interpretation is confirmed by the evolution of the angular distributions. Figure 3b shows the (*p*_*x*_,*p*_*z*_) projections of the 3D-PEELD of fenchone measured by the VMI, as a function of laser ellipticity. The distribution is normalized by the maximum of the 3D-PAD projection: 2(*L* – *R*)/max(*L* + *R*). The PEELD image measured in a quasi-linear field (*S*_3_ = 0.08) is similar in shape to the one measured using *S*_3_ = 0.4, indicating that the photo-selected molecular orientations are similar in the two cases. By extrapolating the linear trend of *G*, we can predict the value of the asymmetry that would be obtained by using circular light (*S*_3_ = 1) to ionize the molecules photoexcited by three linearly polarized photons (dashed line in Fig. 3a): 2.3% in (+)-fenchone, −1.3% in (+)-camphor, and −1.0% in limonene.

When the amount of circularly polarized radiation increases beyond |*S*_3_| ~0.4, circularly polarized photons start to play a significant role in the photoexcitation step, modifying the orientation distributions of photoexcited molecules. This strongly influences the resulting dichroism, inducing a sign change in the asymmetry and a strong increase when reaching circular polarization. Remarkably, the variations of the forward–backward asymmetry in the vicinity of circular polarizations are very sharp: lowering |*S*_3_| from 1 to 0.9 induces a drop of the asymmetry from −2.6% to −0.4%. This is very different from the behavior observed around linear polarization, where increasing |*S*_3_| from 0 to 0.1 only increases the asymmetry by 0.25%. Again we interpret this as the result of the higher efficiency of linearly polarized photons in the multiphoton excitation process. When the polarization state changes from linear to |*S*_3_| = 0.1, we find that the total ionization yield is hardly affected, diminishing by 0.7%. This indicates that the circularly polarized photons are mostly absorbed in the last step of the 3 + 1 REMPI process, and do not influence the orientation distribution of photoexcited molecules. By contrast, when |*S*_3_| decreases from 1 to 0.9, the ionization probability decreases by 40%: the linearly polarized photons play a strong role in the photoexcitation as soon as they are present, sharpening the photoexcitation distribution and modifying the forward–backward asymmetry in electron ejection. This is confirmed by observing the projections of the 3D-PEELD (Fig. 3b): their shape is very different when the polarization state goes from circular to *S*_3_ = 0.9. This interpretation also explains the slope changes observed in 2 + 1 REMPI ionization of camphor at 400 nm^22^: in that case (resonance at 6.2 eV) there was no sign change in the electron asymmetry between molecules excited by linearly and circularly polarized radiation, but the dichroism from the latter was higher than that from the former, leading to a slope increase when |*S*_3_| >0.4.

The resonances involved in our REMPI scheme lie close to the ionization threshold, where the state density is high. Stark shifts can thus occur easily as the laser intensity increases, modifying the states reached by 3 photon absorptions and thus the selected molecular orientations. This appears clearly in Fig. 3c which depicts the PEELD projected distributions on the VMI spectrometer when the intensity is increased to ~1.7 × 10^13^ W cm^−2^. The contribution of the outer ring, associated with 5-photon ionization from the HOMO, strongly increases, but its shape remains quite independent of the ellipticity. The central component, associated with 3 + 1 photon ionization from the HOMO, shows new patterns which reflect the participation of other Stark-shifted intermediate states in the ionization process. Indeed, a strong dependence of the PECD on the intermediate states involved was recently demonstrated in a 2 + 1 REMPI experiment using tunable light^34^. As in the lower intensity case, the shape of the PEELD projection associated with the 3 + 1 ionization from the HOMO is very similar when the amount of circularly polarized light is in the |*S*_3_| <0.4 range, while it dramatically changes between *S*_3_ = 0.9 and 1. This indicates that our conclusions on the relative influence of the circular and linear photons in the excitation process remain valid at higher intensity.

In order to draw a complete picture of the intensity dependence of the PEELD, we investigated the evolution of the variation of the asymmetry factor *G* with ellipticity (Fig. 3d). At the lowest intensity, the asymmetry maximizes when the laser field is elliptical, around *S*_3_ = 0.6. As soon as the intensity reaches a few 10^12^ W cm^−2^, a sharp maximum appears around circular polarization, dominating the asymmetry. When the intensity further increases, the overall asymmetry diminishes, reflecting the transition towards the strong field ionization regime in which the influence of the chiral potential on the electron scattering becomes less important^29^.

### Enantiomeric excess measurement by continuous photoelectron elliptical dichroism

In this section, we use photoelectron elliptical dichroism to determine the enantiomeric excess (*ee*) of a pure chemical compound. Our measurement is based on a continuous and periodic variation of the laser ellipticity, associated with continuous detection of the PEELD (c-PEELD). We sent 515 nm, 2.5 μJ pulses at 2 MHz through a motorized quarter waveplate rotating at 45°/s. The quarter waveplate rotation induced a quasi-sinusoidal modulation of *S*_3_, with a *T*_0_ = 4 s period. The modulated pulses were focused into the VMI to ionize the chiral sample provided by a continuous gas jet connected to flasks containing the samples to analyze. The (*x*,*y*) projection of the 3D photoelectron angular distribution was recorded continuously using a S-CMOS camera with 50 ms acquisition time, acquiring 20 images per second without any dead time. The signal emitted in the forward and backward hemispheres was integrated numerically to extract the instantaneous asymmetry parameter *G*^*raw*^(*t*). In order to correct for the spatial inhomogeneity of the detector’s gain, we averaged the values measured for two opposite helicities by calculating $G(t) = \frac{1}{2}(G^{raw}(t) - G^{raw}(t + T_0/2))$.

**Fig. 4:**
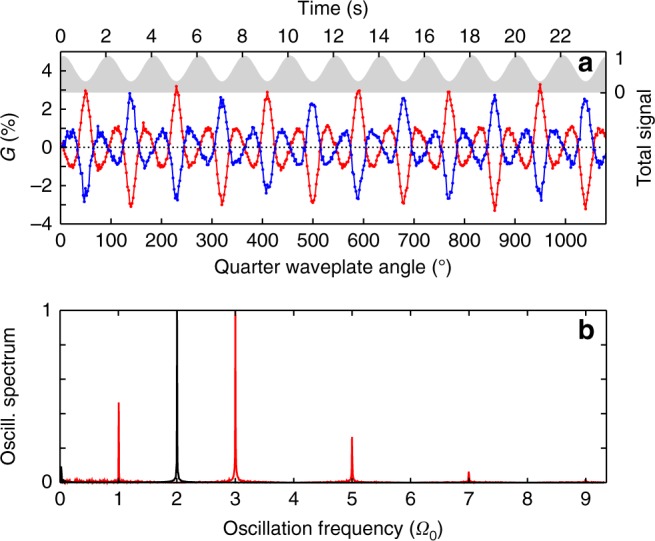
Temporal modulation of PEELD. **a** Temporal evolution of the forward/backward asymmetry *G*(*t*) in (+)-fenchone (red) and (−)-fenchone (blue) at 5 × 10^12^ W cm^−2^ as the ellipticity is continuously scanned by rotating a quarter waveplate. The total photoelectron yield from (+)-fenchone is depicted in gray. **b** Oscillation spectrum of the forward/backward asymmetry (red) and of the total signal (black) obtained by Fourier transforming the temporal signals. The fundamental frequency is Ω_0_ = 0.5 Hz

Figure 4a shows the temporal evolution of the signals measured consecutively in (+) and (−)-fenchone. The total signal maximizes when the laser field is linearly polarized, and minimizes when the field is circular, as seen previously. The modulations of *G*(*t*) follow the variations of *S*_3_, as presented in Fig. 3, and show opposite signs in opposite enantiomers. The high repetition rate of the laser enables a good signal-to-noise ratio to be reached for each data point in 50 ms, corresponding to the sum of 100,000 laser shots. The signal being periodic, it can be decomposed into a sum of frequency components by Fourier transform. Figure 4b shows the fast Fourier transform (FFT) of the signal measured over a 10 min long acquisition. The waveplate rotation speed determines the oscillation frequency of the total signal: it maximizes every 90° of the quarter waveplate, i.e., at 2Ω_0_ = 0.5 Hz, where Ω_0_ = 2*π*/*T*_0_ is the natural fundamental frequency of the experiment corresponding to a quarter waveplate rotation of 180°. The oscillation spectrum of the total signal also shows very weak higher harmonic peaks at *k*Ω_0_, $k \in {\Bbb N}$, reflecting the nonlinearity of the signal variation with *S*_3_ (not visible on the figure scale). The oscillation spectrum of the asymmetry ${\cal G}({\mathrm{\Omega }})$ is much richer, showing peaks at odd harmonics of Ω_0_. This ${\cal G}({\mathrm{\Omega }})$ spectrum reflects the non-linear and non-monotonic dependence of *G* with *S*_3_. It depends on the molecular species, and can be used as a fingerprint of the molecules in multi-component *ee* determination, as we will see later.

The Fourier analysis enables the oscillatory signal to be isolated from noise, acting in a similar manner to a lock-in detection. We will focus on the strongest peak ${\cal G}(3{\mathrm{\Omega }}_0)$, which shows the lowest sensitivity to noise. Since *G*(*t*) is a normalized quantity, the magnitude of this FFT peak $\left| {{\cal G}(3{\mathrm{\Omega }}_0)} \right|$ is proportional to the enantiomeric excess in the sample. Enantiomeric excesses can thus be measured after calibration by a known reference: $ee = \left| {{\cal G}(3{\mathrm{\Omega }}_0)} \right|/\left| {{\cal G}^{ref}(3{\mathrm{\Omega }}_0)} \right| \ast ee^{ref}$. Here we used enantiopure (+)-fenchone molecules as a reference, assuming $ee^{ref} = ee^{(+)} = 1$, but a mixture with a known enantiomeric excess could as well be used.

We measured the composition of three samples, using a 10 min acquisition for each. A detailed analysis of the measurement errors is presented in the Methods section. The first sample was a pure (−)-fenchone sample from Sigma Aldrich, whose enantiomeric excess is specified as *ee* = −84.2 ± 4%. Our measurements provide a more accurate determination of this value. Next we composed two mixtures using a precision balance. In all cases, the c-PEELD measurements provide high accuracy determination of the *ee*, as shown in Table 1.

**Table 1:** Measurement of the enantiomeric excess of three mixtures of fenchone using (+)-fenchone as a reference with $ee^{ref} = 1$

Mixture	Enantiomeric excess from provider	Enantiomeric excess measured by c-PEELD
(−)-fenchone	−84.2 ± 4%	−84.0 ± 0.4%
Mix 1	63.1 ± 4%	62.7 ± 0.5%
Mix 2	25.1 ± 4%	24.9 ± 0.4%

Each measurement corresponds to a 10 min acquisition

The error bars of the c-PEELD measurements are 95% confidence intervals (see Methods)

One important advantage of c-PEELD measurements is their continuous nature and the absence of dead time in the acquisition. By contrast, conventional PECD measurements rely on the detection of photoelectrons produced by left and right circularly polarized light, which is obtained by a 90° quarter waveplate rotation in laser-based experiments. With kHz lasers, the time taken to rotate the waveplate is negligible compared to the acquisition time necessary to build up the statistics on the asymmetric factor *G*. However this is far from the case when MHz lasers are used, the acquisition time being 50 ms in the present experiment. Even using very fast direct-drive motorized rotation stages, the acceleration and deceleration times set a limit of a few 100 ms for the switching time between the two helicities, imposing a significant dead time in the acquisition. Alternative solutions exist, such as electro-optic modulators, but their use is much more cumbersome than a rotation stage. Furthermore, the continuous rotation used in c-PEELD optimizes the acquisition process by using the whole ellipticity dependence of the photoelectron asymmetry.

**Fig. 5:**
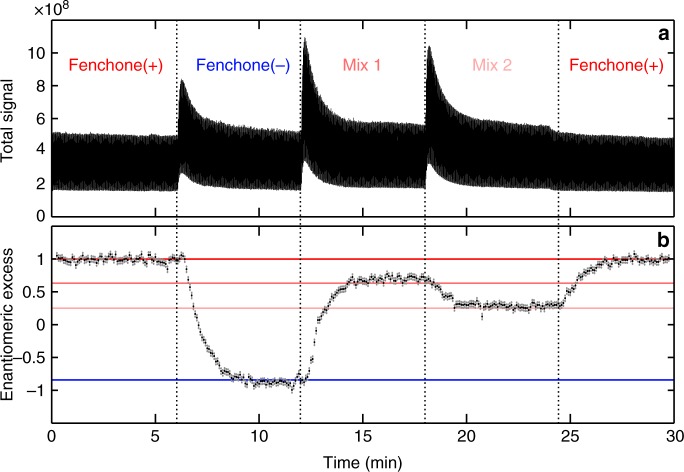
Continuous monitoring of enantiomeric excess by c-PEELD. **a** Total photoelectron signal as a function of time, as different samples are successively connected to the chamber. **b** Measured enantiomeric excess (black dots) and 95% confidence error bars (gray). The horizontal lines are the theoretical values of the enantiomeric excesses of the different mixtures

To demonstrate the possibility of monitoring enantiomeric excesses in real time, we performed a dynamical measurement. We connected four flasks, containing (+)-fenchone, (–)-fenchone, and the two mixtures measured above, to the gas line supplying the gas jet. We successively opened the different flasks, while keeping the others closed, and recorded the c-PEELD measurements on-the-fly. The evolution of the total photoionization signal is shown in Fig. 5a. The pressure increases every time a new flask is connected to the chamber, causing an increase of the signal, which relaxes afterwards in a few tens of seconds. The photoelectron asymmetry *G*(*t*) was extracted from the data and analyzed by Gabor analysis, using a Gaussian window of 5 s full width at half maximum (FWHM) duration. The instantaneous enantiomeric excess *ee*(*t*) was obtained by measuring the magnitude of the FFT peak $\left| {{\cal G}(3{\mathrm{\Omega }}_0)} \right|$, while its sign was extracted from the phase of ${\cal G}(3{\mathrm{\Omega }}_0)$ (when the enantiomeric excess switches sign, the phase of the oscillations of *G*(*t*) shifts by *π*, as seen in Fig. 4a). Figure 5b presents the evolution of the enantiomeric excess in the VMI chamber as a function of time. Each flask switching causes a transient in *ee*(*t*), with a typical timescale of a few minutes, characteristic of the gas dynamics in the tubes towards the VMI chamber. After this transient, the enantiomeric excess tends to the expected value, within the ±4% error bar determined by the duration of the Gabor window (see Methods). The slight systematic offset of the measured values for Mix 1 and Mix 2 could be due to a drift of laser intensity (see Methods). The continuous locking of the laser power and duration should further improve the accuracy of the c-PEELD measurements. This experiment demonstrates the ability to follow the enantiomeric composition of a gaseous sample in real time, with a temporal resolution of a few seconds and an accuracy of a few percent. This constitutes a major breakthrough for chiral analysis in the gas phase.

**Fig. 6:**
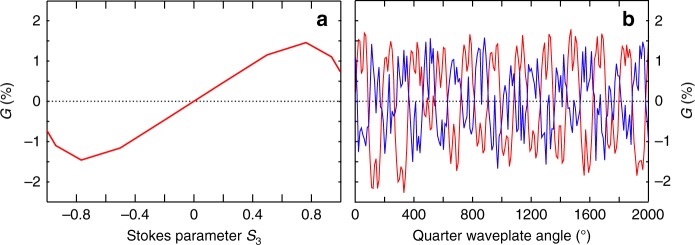
PEELD measurement with the CERSEI instrument. Evolution of the forward/backward asymmetry *G* in (+)-fenchone (red) and a fenchone mixture with *ee* = −63% using 520 nm pulses at ~1 × 10^12^ W cm^−2^, as a function of S_3_ (**a**) and as the ellipticity is continuously scanned by rotating a quarter waveplate (**b**)

While the measurements presented above used a COLTRIMS or a VMI, two rather costly instruments, a simpler device can be used to perform c-PEELD measurements for direct real-time analysis. At Queen's University Belfast, an instrument has been constructed so that *G* can be measured directly by collecting the electron yield in the forward and backward hemispheres, whatever their kinetic energy^22^ (see Methods). Figure 6a shows the measured forward/backward asymmetry measured with this instrument, using 520 nm pulses at ~1 × 10^12^ W cm^−2^, with a repetition rate of 1 MHz. The ellipticity dependence of *G* is consistent with the low intensity measurements presented in Fig. 3d, with a strong change in *G* as *S*_3_ approaches 1 but without a change in sign. Enantiomeric excesses can still be extracted from Fourier analysis of a c-PEELD signal *G*(*t*). We obtain a value of *ee* = −63 ± 5% for a mixture previously determined as *ee* = −66%. Using higher laser intensity would strongly increase the signal and its non-linear dependence on *S*_3_, leading to an improvement of the accuracy. Nevertheless, this measurement demonstrates the possibility of using a very simple and compact instrument to perform PEELD and c-PEELD measurements.

### Analysis of multi-component samples

We have focused until now on the analysis of samples containing only one chiral species. The enantiomeric analysis of multi-component mixtures is a challenging task. Recently, Fanood et al.^23^ showed that mass-tagged PECD offers an interesting solution to this problem. They detected in coincidence the photoions and photoelectrons produced by circularly polarized femtosecond laser pulses. This enabled them to resolve the PECD associated to each ion, and to distinguish different molecular species. They managed to measure the enantiomeric excess of limonene and fenchone in a mixture. While mass-spectrum resolved PECD is undoubtedly a powerful analytical technique, it suffers from long acquisition times imposed by the coincidence detection. Here we show that c-PEELD offers a valuable alternative.

The key idea behind multi-component analysis with c-PEELD is to use the ellipticity dependence of the photoelectron signal as a fingerprint of the molecules. As we have seen in Fig. 3, the forward–backward asymmetry in the electron ejection can be very different from one molecule to another. For instance, the asymmetry maximizes in circularly polarized light in fenchone and in elliptical light in camphor. This means that these two species will show different oscillation spectra in a c-PEELD measurement. On the other hand, two different species can show very similar behaviors and would not be distinguished (for instance, camphor and limonene). Such ambiguities can be lifted by increasing the dimensionality of the measurement.

Photoelectron elliptical dichroism produces characteristic 3D photoelectron angular distributions, which can be measured by COLTRIMS or VMI tomography. These 3D maps carry very rich signatures of the ionized species (Fig. 2), and could certainly be used for multi-component mixture analysis. However, their acquisition time is incompatible with real-time monitoring, since it requires electron counting (COLTRIMS) or recording multiple projections with a VMI. On the other hand, the c-PEELD analysis shown up to now was focused on the forward–backward electron asymmetry *G*(*t*), which is an angle- and energy-integrated quantity. A tradeoff can be found by analyzing the (*x*, *z*) projections of both the 3D-PAD and 3D-PEELD, which can be measured in a VMI in a few tens of milliseconds.

In order to generate molecular fingerprints, we analyze the temporal oscillations of the projections of the 3D electron distributions on the VMI detector as the ellipticity is continuously scanned: $\overline P (p_x,p_z,t) = {\int} P(p_x,p_y,p_z,t)dp_y$. The forward/backward symmetric part of the image $\overline {P} ^{sym}(p_x,p_z,t) = \frac{1}{2}\left( {\overline P (p_x,p_z,t) + \overline P (p_x, - p_z,t)} \right)$ provides the evolution of the projection of the PAD. The antisymmetric part $\overline P ^{anti}(p_x,p_z,t) = \frac{1}{2}\left( {\overline P (p_x,p_z,t) - \overline P (p_x, - p_z,t)} \right)$ is the chiral-sensitive projection of the PEELD signal. Each pixel of the detector plane (*x*, *z*) is analyzed by Fourier transform to obtain the oscillation spectra $\overline {\cal P} ^{sym}(p_x,p_z,{\mathrm{\Omega }})$ and $\overline {\cal P} ^{anti}(p_x,p_z,{\mathrm{\Omega }})$. These spectra respectively show peaks at frequencies 2*k*Ω_0_ and (2*k* + 1)Ω_0_, $k \in {\Bbb N}$, as observed in the analysis of the integrated signal ${\cal G}({\mathrm{\Omega }})$ (Fig. 4). The symmetric and antisymmetric signals are respectively even and odd with respect to variations of the Stokes parameter *S*_3_, and thus with respect to the quarter waveplate rotation. As a consequence, their oscillation spectra are respectively purely real and imaginary.

**Fig. 7:**
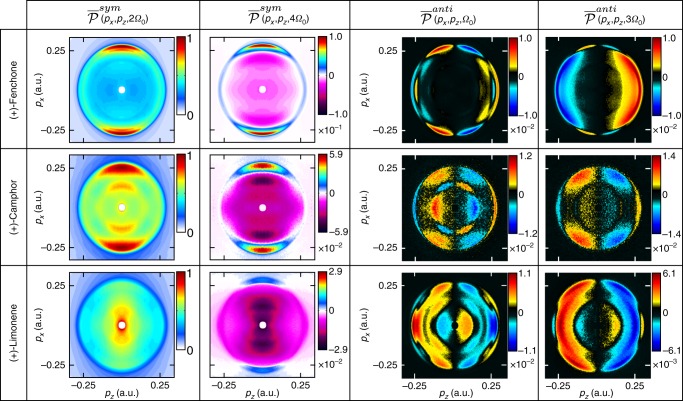
Molecular fingerprints in PEELD measurements. Fourier decomposition of the oscillations of the VMI images with ellipticity in (+)-fenchone (top), (+)-camphor (mid) and (+)-limonene (bottom), using 515 nm pulses at 5 × 10^12^ W.cm^−2^. Left: Symmetric components of the image $\overline {\cal P} ^{sym}(p_x,p_z,{\mathrm{\Omega }})$. Right: Antisymmetric components of the images $\overline {\cal P} ^{anti}(p_x,p_z,{\mathrm{\Omega }})$. All values are normalized to the maximum of $\overline {\cal P} ^{sym}(p_x,p_z,{\mathrm{\Omega }}_0)$. The *z*-axis is the laser propagation direction and the *x*-axis is the main axis of the laser polarization ellipse

Figure 7 shows the amplitude of the different peaks of the oscillation spectra of the projected PAD and PEELD images, measured in fenchone, camphor and limonene. The three molecular species clearly show different two-dimensional (2D) fingerprints which could be used for the analysis of chiral mixtures. The spectroscopic assignment of the structures observed on these fingerprints is beyond the scope of this paper. Let us simply mention that the sign changes in the low-energy range PEELD can result from vibrational excitation of the ion, as observed in PECD^29,35,36^, but also from low-energy electron scattering. However, one-dimensional (1D) fingerprints are indeed enough to measure the composition of a multi-component mixture, offering the opportunity to perform faster analysis for real-time monitoring. To obtain 1D distributions, we project the 2D VMI images along the laser propagation direction: $\overline {\overline P } (p_z,t) = {\int} \overline P (p_x,p_z,t)dx$. We calculate the symmetric and antisymmetric components, Fourier transform them, and finally get the 1D fingerprints $\overline {\overline {\cal P} } ^{sym}(p_z,2k{\mathrm{\Omega }}_0)$ and $\overline {\overline {\cal P} } ^{anti}(p_z,(2k + 1){\mathrm{\Omega }}_0)$.

**Fig. 8:**
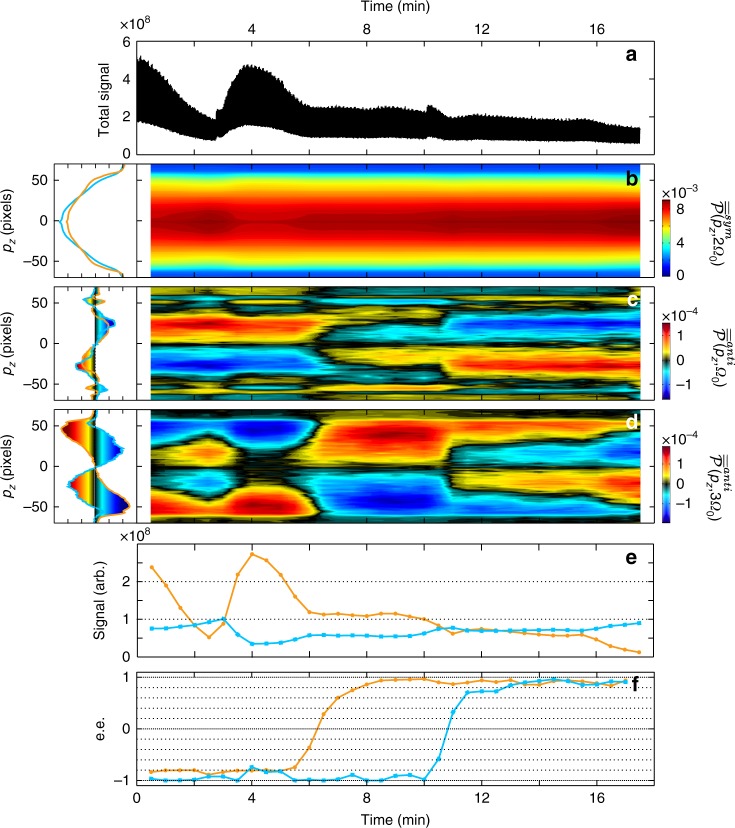
Real-time analysis of a dynamical camphor-fenchone mixture. The fingerprints $\overline {\overline {\cal P} } ^{sym}(p_z,2{\mathrm{\Omega }}_0)$, $\overline {\overline {\cal P} } ^{anti}(p_z,{\mathrm{\Omega }}_0)$ and $\overline {\overline {\cal P} } ^{anti}(p_z,3{\mathrm{\Omega }}_0)$ from pure (+)-fenchone (orange) and (+)-camphor (blue) were recorded using pure samples before the dynamical measurement and are presented in the left column. At *t* = 0, two flasks of (−)-fenchone and (−)-camphor were connected to the gas jet. At *t* = 2′30″, the valve openings were adjusted to increase the fenchone contribution. At *t* = 5 min, the (−)-fenchone flask was closed and a (+)-fenchone flask was opened. At t = 10 min, the (−)-camphor was closed and a (+)-camphor flask was opened. **a** Total signal from the gas mixture during the c-PEELD measurement. **b** Temporal evolution of the 1D symmetric component at 2Ω_0_, extracted from a Gabor analysis with a 30 s window. **c** 1D antisymmetric component at Ω_0_. **d** 1D antisymmetric component at 3Ω_0_. **e** Contribution to the total signal and **f** enantiomeric excess of fenchone (orange) and camphor (blue) retrieved by fitting the symmetric and antisymmetric components

After determining the 1D fingerprints from pure fenchone and camphor (left column in Fig. 8), we performed a c-PEELD measurement in a mixture of these two compounds. We connected flasks containing enantiopure (+)- and (−)-camphor and fenchone to the gas jet, and alternatively opened them to continuously vary the composition of the VMI target. The VMI images were measured on-the-fly as the quarter waveplate rotated continuously, with the same parameters as in previous measurements. To extract the time-resolved composition of the samples, we use a Gabor analysis with a Gaussian filter of 30 s FWHM, and calculate $\overline {\overline {\cal P} } ^{sym}(p_z,2k{\mathrm{\Omega }}_0)$ and $\overline {\overline {\cal P} } ^{anti}(p_z,(2k + 1){\mathrm{\Omega }}_0)$ as a function of the central position of the filter. The symmetric part $\overline {\overline {\cal P} } ^{sym}(p_z,2{\mathrm{\Omega }}_0)$ (Fig. 8c) is used to extract the relative proportions of fenchone and camphor and their contribution to the signal (Fig. 8e). These contributions take into account differences in partial pressure and ionization probabilities. The latter could be normalized out by calibration in pure samples at the same pressure. The two main frequency components of the antisymmetric part $\overline {\overline {\cal P} } ^{anti}(p_z,{\mathrm{\Omega }}_0)$ and $\overline {\overline {\cal P} } ^{anti}(p_z,3{\mathrm{\Omega }}_0)$(Fig. 8c, d) are used to determine the enantiomeric excess of each species (see Methods).

We started with a situation where both (−)-camphor and (−)-fenchone were connected to the target tube through partly open valves. From *t* = 0 to *t* ≈ 2′30″ the total signal decreased. Figure 8e reveals that this was due to a drop of the fenchone pressure. At *t* = 2′30″ we increased the (−)-fenchone valve opening and slightly closed the (−)-camphor valve, increasing the total signal (Fig. 8a) and the fenchone contribution (Fig. 8b). During this phase, the instantaneous enantiomeric excess measurements give *ee*_fenchone_ = −82 ± 5% and *ee*_camphor_ = −94 ± 2% (95% confidence interval). At *t* = 5 min, we switched the fenchone sample, from (−) to (+)-fenchone. The c-PEELD measurements clearly detects this transition and shows that it occurs in about 3 min. From *t* = 9 min to the end of the measurement, the enantiomeric excess of fenchone is *ee*_fenchone_ = 91 ± 8%. At *t* = 10 min, we switched the camphor enantiomer. Again, the *ee* measurement tracks the dynamics of this change and ends up with the right enantiomeric composition. We observe a continuous decrease of the fenchone pressure from 5 min to the end of the measurement, but this does not affect the *ee* measurement. The c-PEELD technique is thus able to track variations of the composition and *ee* in a mixture, offering excellent accuracy and temporal resolution. Note that this technique is able to distinguish two compounds that have the same mass, such as fenchone and camphor, which would be difficult using a mass spectrometer.

## Discussion

Photoelectron elliptical dichroism in multiphoton ionization is a remarkably rich phenomenon. Whereas one-photon PECD scales linearly with the amount of circular polarization, the PEELD measured in REMPI can exhibit very abrupt variations with *S*_3_. These variations reflect the competition between linearly and circularly polarized photons in the photoexcitation process, which determines the anisotropy of the photoexcited molecular ensemble. From a fundamental point of view, extrapolating the linear behavior of PEELD measurements near *S*_3_ = 0 to *S*_3_ = 1 provides an interesting physical quantity—the photoelectron circular dichroism that would be measured by photoexciting molecules with linear photons and ionizing them with circular photons—which can be particularly useful to benchmark calculations of REMPI-PECD^32,37,38^. In the same spirit, the 3D-PEELD distributions that we have measured exhibit very characteristic features, depending on the molecular species, and are certainly very sensitive probes of the quality of quantum chemistry calculations in chiral molecules. From a more applied point of view, continuous PEELD measurements prove to be an extremely powerful probe of chiral samples. This accuracy results from the Fourier analysis of the continuously recorded data, in which the abrupt variations of PEELD with *S*_3_ produce characteristic frequency peaks. This analysis does not require quantum-chemical calculation as it is often the case for *ee* determination by microwave spectroscopy^9^. Enantiomeric excesses from mono-component samples can be determined with accuracies of 0.4% in 10 min and 5% in 3 s, enabling real-time monitoring. In multi-component samples, the instantaneous composition can be followed, with an accuracy of a few percent and a temporal resolution of 30 s. A remaining point to investigate is the possible limitations encountered when components with very different vapor pressures or ionization probabilities need to be identified. In that case it is probable that scanning the laser wavelength could provide a way to balance the signals from different species by optimizing the resonance transitions in the compound giving the lowest signal. Indeed, the wavelength dependence could provide additional discrimination, especially if picosecond pulses with a narrower bandwidth are used. However, even without exploiting the full dimensionality of the current data, and given the relative simplicity of the setup and data processing, we believe that this method could become a standard in analytical chemistry.

## Methods

### Laser system at CELIA

All experiments (except Fig. 6) were conducted using the Blast Beat laser system at CELIA consisting of two optically synchronized industrial ultrafast fiber amplifiers seeded by the same oscillator, each delivering 130 fs pulses at 1030 nm with 50 W average power and a tunable repetition rate from 166 kHz to 2 MHz (Tangerine Short Pulse, Amplitude Systemes).

### COLTRIMS measurements

The 515 nm laser pulses were focused by a *f* = 50 cm lens with a numerical aperture ~0.08 in the interaction chamber of the Cold Target Recoil Ion Momentum Spectrometer (COLTRIMS, RoentDek GmbH). Enantiopure fenchone molecules (Sigma Aldrich) at room temperature were carried under vacuum by a gas line heated at 80°C into a 30 μm nozzle heated at 120°C. The gas jet was seeded by 1.0 bar of argon. A 200 μm diameter skimmer, placed 9 mm after the nozzle, was used to select the most collimated part of the gas jet. The ions were accelerated towards a set of dual microchannel plates and collected using a delay line anode, which measured their position and arrival time. The signal was isolated using RC decouplers, amplified, and a Constant Fraction Discriminator was used to remove the amplitude dependency before digitalization of the timings. As the ionization potential of argon (15.76 eV) is much higher than that of fenchone (8.72 eV), no argon ions were detected. The electrons were guided by a homogeneous magnetic field and accelerated by an electric field towards a set of dual microchannel plates (MCPs) and an hexanode. Since the ion signal was dominated by fenchone, we did not perform any electron-ion coincidence and detected all the produced electrons. The laser repetition rate was adjusted between 400 kHz and 1 MHz to ensure a constant count rate of 100 kHz on the detector when the ellipticity was varied, while keeping the same pulse energy. The delay line anodes enable the full 3D momentum distribution of the ejected photoelectrons to be measured. For each laser ellipticity, measurements were taken by alternating positive and negative helicity every 3 × 10^7^ detected electrons, to reach a total number of electrons of 3 × 10^8^. The total acquisition duration for the data shown in Fig. 1 was less than 1 h.

### Velocity map imaging measurements

The laser polarization state was controlled by a continuously rotating direct-drive stage, and the acquisition was triggered by the stage to ensure that the absolute phase of the c-PEELD oscillations remained unchanged from one measurement to the next. The samples were provided by 5 flasks lying in a water bath heated at 40°C and connected to the jet by a 60 cm long stainless steel tube with 4 mm inner diameter. The tube was heated to 120°C and a glass fiber filter was used to avoid the formation of droplets in the jet. No seed gas was used. The continuous gas jet (250 μm nozzle, 40 mm away from a 2 mm skimmer) was directed towards the interaction zone of the VMI, where a set of electrodes projected the 3D photoelectron distributions onto a double stack of MCPs, imaged by a phosphor screen. A S-CMOS camera measured the 2D images of the projected photoelectron distribution, with 50 ms acquisition time and 20 images per second (no dead time between two images). Each 3D-PEELD map shown in Fig. 2 was typically recorded in 15 min.

### Belfast’s measurements

**Fig. 9:**
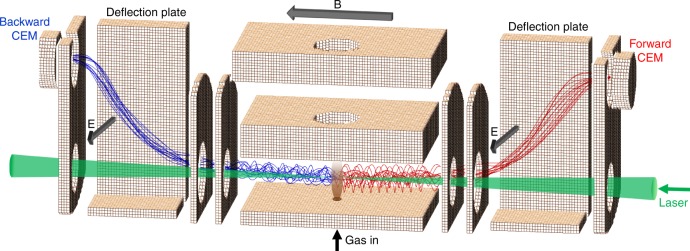
Schematic of the Belfast CERSEI instrument used to directly measure PEELD. Femtosecond laser pulses were focused into a vacuum chamber through a series of apertures into an effusive gas jet. The gas jet emerged from a capillary in one of the grounded plates which sandwich the interaction region. A pair of current-carrying coils is used to generate a magnetic field (B) of around 30 Gauss parallel to the laser beam. As a result, any photoelectrons emitted from multiphoton ionization of the gas spiral along the field lines allowing those emitted in the forward (blue) and backward (red) directions to spatially separate. Pairs of parallel deflection plates (one plate of the pair is cut away for clarity in the figure) are used to generate an electric field (E) which separates each bunch of electrons from the laser and directs them onto separate channel electron multipliers (CEM). The electrodes and simulated trajectories were produced by SIMION 8.0 charge particle optics software^39^. The CEM detectors are separated by 130 mm and the instrument sits inside a vacuum chamber with an outer footprint of 300 × 200 × 150 mm

Measurements at Queen’s University Belfast (Fig. 6) were conducted using a device dubbed CERSEI (Chiral Electron Removal and Separation for Enantiomer Identification), which directly measures the number of electrons emitted forward and backward. Rather than using an electric field to project the electrons onto a detector, a magnetic field (of around 30 Gauss) is generated parallel to the laser propagation by two current-carrying coils. This keeps the photoelectrons confined along the laser axis so that the direction in which they drift is determined by their initial emission angle—forward if less than 90°, backward if greater than 90°. The number of electrons in these two groups can then be counted separately in two channel electron multipliers (CEMs) via the stereo-detection scheme shown in Fig. 9. In the present experiment, the detectors were operating in counting mode, with a count rate below 0.1 event per laser pulse to avoid saturation effects. The measurements speed could be significantly increased if MCPs were used instead.

As the detectors need to be offset from the laser beam, two pairs of deflection plates are used to deflect the electrons into the CEMs using an E × B field. The average potential of these plates is positive so that the electrons are accelerated as well as deflected. This ensures that the trajectories of all the electrons emitted in each direction lie within the solid angle of their respective detector, regardless of the initial emission angle or energy.

The target molecules were introduced into the interaction region via a room temperature effusive gas jet which emerged from a capillary in one of the plates comprising the interaction region. For the measurements, a Spectra Physics Spirit HE laser generating 300 fs pulses at repetition rate of 1 MHz was coupled to the device. Second harmonic pulses at 520 nm and an energy of 1.8 μJ were focused with a 20 cm focal length lens into the center of the gas jet to produce peak intensities approaching 10^12^ W cm^−2^. The small chamber was pumped by a 200 L s^−1^ turbopump to less than 10^−7^mbar which increased to around 10^−6^mbar during measurements.

The laser polarization state was controlled by the angle of a quarter waveplate placed just before the focussing lens. During measurements, data were acquired for 30 s at 10° intervals of the waveplate angle. In order to account for any inequality in the detection efficiency of the two CEMs and small drifts of laser power or target density, the value of *G* was calculated using two measurements corresponding to polarization states with the same ellipticity but opposite helicity $G(t) = \frac{1}{2}\left( {G^{raw}(t) - G^{raw}(t + T_0/2)} \right)$.

### Error analysis in c-PEELD measurements

**Fig. 10:**
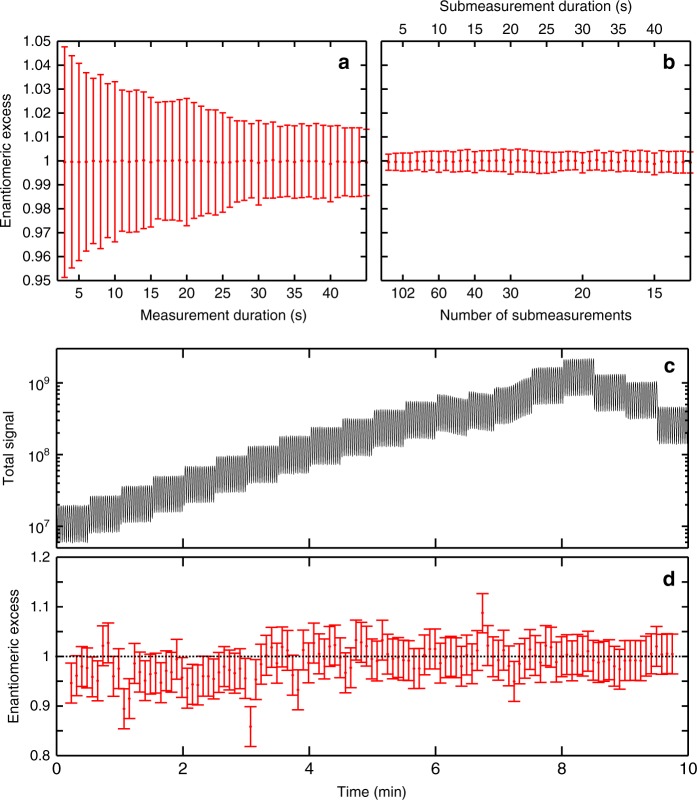
Statistical analysis of the enantiomeric excess measurement by PEELD. **a** The 95% confidence interval of the enantiomeric excess of a pure (+)-fenchone sample, as a function of the duration of the measurement. **b** Results of a 10 min *ee* measurement by statistical analysis of sets of submeasurements, as a function of the number and duration of the submeasurements. **c**, **d** Total signal and enantiomeric excess measured while increasing the gain of the microchannel plate detector every 30 s

We first analyze the statistical error in the determination of the enantiomeric excess as a function of the measurement duration. We cut a 10 min measurement in (+)-fenchone into successive slices by applying a Gaussian filter of duration *τ* (FWHM). We measure the enantiomeric excess from each slice, and perform a statistical analysis of the results. Figure 10a shows the 95% confidence interval on a measurement as a function of its duration. The enantiomeric excess can be determined with a 5% accuracy in 3 s. Increasing the measurement duration to 30 s enables reaching accuracies in the 2% range, but the uncertainty saturates after that. This means that to further improve the measurements, we should repeat it several times and average the results. Figure 10b shows the results of this procedure. The total measurement time is 10 min but the measurement is split into a decreasing number of submeasurements of increasing duration. The 95% error bar remains below 0.5% whatever the subset chosen, but choosing a large number of short submeasurements is favorable, enabling a 0.3% accuracy to be reached. Note that the symmetrization procedure $G(t) = \frac{1}{2}(G^{raw}(t) - G^{raw}(t + T_0/2))$ is absolutely not necessary for the chiral analysis: the artifacts in the detection appear as other frequency components in the analyzed signal, such that the same results are found using *G*(*t*) and *G*^*raw*^(*t*), with the same accuracy.

Beyond this statistical analysis, one can wonder about the origin of systematic errors in the measurement. First, a systematic error in the *ee* measurement by c-PEELD can be introduced by the calibration step. The enantiomeric excesses are determined with respect to a reference sample, whose enantiomeric purity must be known accurately. Furthermore, the reference measurement will introduce an inherent uncertainty but this can be minimized by performing a very long acquisition. Second, since the enantiomeric excess is determined by the measurement of a modulation amplitude, the linearity of the detector is an important parameter. To estimate its impact on the measurements, we performed a 10 min acquisition in enantiopure (+)-fenchone, and increased the voltage on the microchannel plates by 20 V every 30 s, then decreased it again. The level of the detected signal varied by two orders of magnitude during this procedure. We ran a Gabor analysis of the results using a Gaussian window with 3 s FWHM, and measured the temporal variation of the peak $I_{3{\mathrm{\Omega }}_0}$ to determine the instantaneous enantiomeric excess (Fig. 10c, d). A systematic error is clearly present over the first 3 min: the *ee* values are systematically lower than the actual value. However, the discrepancy is not large, around 5%, i.e., within most statistical error bars. This means that for the lowest signal value, a new calibration of $I_{3{\mathrm{\Omega }}_0}^{ref}$ should be performed. This result demonstrates however that *ee* can be faithfully measured over more than one order of magnitude of signal variations, such as the ones that could result from molecular density variations. The other important source of statistical error is drifts of the laser intensity. As we have seen in the previous section, PEELD is indeed very sensitive to laser intensity. This means that once the calibration is done, the laser intensity should be kept constant. Fiber laser systems are intrinsically very stable, but they deliver a high average power which has to be dealt with adequately in order to avoid thermal drifts of the beam properties.

### Multi-component mixture analysis

To obtain 1D distributions, we project the 2D VMI images along the laser propagation direction: $\overline {\overline P } (p_z,t) = {\int} \overline P (p_x,p_z,t)dx$. We calculate the symmetric and antisymmetric components, Fourier transform them, and finally get the 1D fingerprints $\overline {\overline {\cal P} } ^{sym}(p_z,2k{\mathrm{\Omega }}_0)$ and $\overline {\overline {\cal P} } ^{anti}(p_z,(2k + 1){\mathrm{\Omega }}_0)$. Once the fingerprints from pure compounds have been determined, we perform a c-PEELD measurement in a mixture of two compounds A and B to extract $\overline {\overline {\cal P} } _{mix}^{sym}(p_z,2k{\mathrm{\Omega }}_0)$ and $\overline {\overline {\cal P} } _{mix}^{anti}(p_z,(2k + 1){\mathrm{\Omega }}_0)$. A least-square algorithm is used to minimize the function

1 $$\begin{array}{*{20}{l}} {f = \left| {\overline {\overline {\cal P} } _{mix}^{sym}(p_z,2{\mathrm{\Omega }}_0) - a\overline {\overline {\cal P} } _A^{sym}(p_z,2{\mathrm{\Omega }}_0) - (1 - a)\overline {\overline {\cal P} } _B^{sym}(p_z,2{\mathrm{\Omega }}_0)} \right|^2} \hfill \\ { + \alpha _4\left| {\overline {\overline {\cal P} } _{mix}^{sym}(p_z,4{\mathrm{\Omega }}_0) - a\overline {\overline {\cal P} } _A^{sym}(p_z,4{\mathrm{\Omega }}_0) - (1 - a)\overline {\overline {\cal P} } _B^{sym}(p_z,4{\mathrm{\Omega }}_0)} \right|^2} \hfill \\ { + \alpha _6\left| {\overline {\overline {\cal P} } _{mix}^{sym}(p_z,6{\mathrm{\Omega }}_0) - a\overline {\overline {\cal P} } _A^{sym}(p_z,6{\mathrm{\Omega }}_0) - (1 - a)\overline {\overline {\cal P} } _B^{sym}(p_z,6{\mathrm{\Omega }}_0)} \right|^2} \hfill \\ { + ...} \hfill \end{array}$$

where *a* and (1 − *a*) are the relative contributions of components *A* and *B* to the ionization signal, and *α*_*j*_ are coefficients used to adjust the relative weight of the different frequency components *j*Ω_0_, $j \in 2{\Bbb N}_{ > 1}$. In the present case we only used the first Fourier peak and set all *α*_*j*_ to zero. Getting a calibration of the signal level with respect to the pressure can be done separately with pure compounds, so that in the mixture the fingerprint measurement of *a* (and hence 1 − *a*) and the total integrated level provide absolute measurement of the partial pressures of *A* (and *B*), in a non enantiomer-specific manner at this stage.

Once the contributions of the two compounds are determined, the projected PEELD signal can be used to measure the enantiomeric excesses of the two compounds *ee*_*A*_ and *ee*_*B*_ by minimizing the function:

2 $$\begin{array}{*{20}{l}} {g = \left| {\overline {\overline {\cal P} } _{mix}^{anti}(p_z,{\mathrm{\Omega }}_0) - a.ee_A.\overline {\overline {\cal P} } _A^{anti}(p_z,{\mathrm{\Omega }}_0) - (1 - a).ee_B.\overline {\overline {\cal P} } _B^{anti}(p_z,{\mathrm{\Omega }}_0)} \right|^2} \hfill \\ { + \beta _3\left| {\overline {\overline {\cal P} } _{mix}^{anti}(p_z,3{\mathrm{\Omega }}_0) - a.ee_A.\overline {\overline {\cal P} } _A^{anti}(p_z,3{\mathrm{\Omega }}_0) - (1 - a).ee_B.\overline {\overline {\cal P} } _B^{anti}(p_z,3{\mathrm{\Omega }}_0)} \right|^2} \hfill \\ { + \beta _5\left| {\overline {\overline {\cal P} } _{mix}^{anti}(p_z,5{\mathrm{\Omega }}_0) - a.ee_A.\overline {\overline {\cal P} } _A^{anti}(p_z,5{\mathrm{\Omega }}_0) - (1 - a).ee_B.\overline {\overline {\cal P} } _B^{anti}(p_z,5{\mathrm{\Omega }}_0)} \right|^2} \hfill \\ { + ...} \hfill \end{array}$$

where *β*_*j*_ are coefficients used to adjust the relative weight of the different frequency components *j*Ω_0_, $j \in 2{\Bbb N}_{ > 1} - 1$. Here we set a weight of 10 to *β*_3_ and set higher components to zero, to put emphasis on the dominant peak in the signal, which was less sensitive to noise. The minimization procedure can be sequential, first determining *a* by minimizing *f* and then *ee*_*A*,*B*_ by minimizing *g*, or globally through the minimization of a weighted sum of *f* and *g*. In the present case we obtained similar results with the two procedures. The partial pressure of each enantiomer of each compound is then accessible using the pressure calibration mentioned above.

## Data Availability

The datasets generated and analyzed during the current study are available from the corresponding author on reasonable request.
